# Antigenicity and infectivity of severe acute respiratory syndrome coronavirus 2 Omicron subvariants EG.5.1, XBB.2.3, FL.1.5.1, and BA.2.86

**DOI:** 10.1002/mco2.589

**Published:** 2024-06-08

**Authors:** Haijun Tang, Yanhang Zhuo, Xiaohong Du, Frank Xiao‐Feng Qin, Yi Huang

**Affiliations:** ^1^ Shengli Clinical Medical College of Fujian Medical University Fuzhou China; ^2^ Center for Experimental Research in Clinical Medicine Central Laboratory Fujian Provincial Hospital Fuzhou China; ^3^ National Key Laboratory of Immunity and Inflammation Suzhou Institute of Systems Medicine Chinese Academy of Medical Sciences & Peking Union Medical College Suzhou China

Dear Editor,

The continuous mutation of severe acute respiratory syndrome coronavirus 2 (SARS‐CoV‐2) within the ongoing epidemic poses a formidable challenge to public health.^1^, ^2^ Following the emergence of Omicron BA.1, the rate of virus evolution has notably accelerated, giving rise to numerous new Omicron subvariants.^1^, ^3^ Currently, BA.2.86 and its descendant JN.1 are predominant, harboring over 30 additional spike protein mutations compared to the BA.2 strain.^3^ These mutations in BA.2.86 and JN.1 may enhance transmissibility, potentially increasing re‐infection risks. The infection characteristics, cross‐species propensities, membrane fusion response, and immune evasion capability of these newly emerged variants need to be systematically explored to provide new strategies for the prevention and control of coronavirus disease 2019.

To investigate the infectivity of the major circulating Omicron strains, we prepared each pseudovirus using the vesicular stomatitis virus pseudovirus system. Pseudoviruses carrying spike proteins of BA.4/5, XBB, XBB.1.5, EG.5, EG.5.1, and BA.2.86 exhibited higher infectivity than BA.2 (Figure 1A, left panel). In contrast, XBB.1.16, XBB.2.3, and FL.1.5.1 showed similar or decreased infectivity. We also explored the susceptibility of cells expressing angiotensin‐converting enzyme 2 (ACE2) homologs from 10 species to Omicron subvariants. All examined Omicron subvariants efficiently infected these cells, albeit with varying efficiencies (Figure 1A, right panel). Notably, cells expressing human, camel, and pig ACE2 showed greater susceptibility to Omicron subvariants than those expressing ferret ACE2. Moreover, D614G, BA.2, and BA.286 pseudoviruses manifested lower infectivity against cells with ACE2 from horses, rats, mice, cats, and dogs, whereas other Omicron strains showed enhanced infectivity. These observations indicate an evolving trend in Omicron subvariants towards heightened cellular infectivity across species.

**FIGURE 1:**
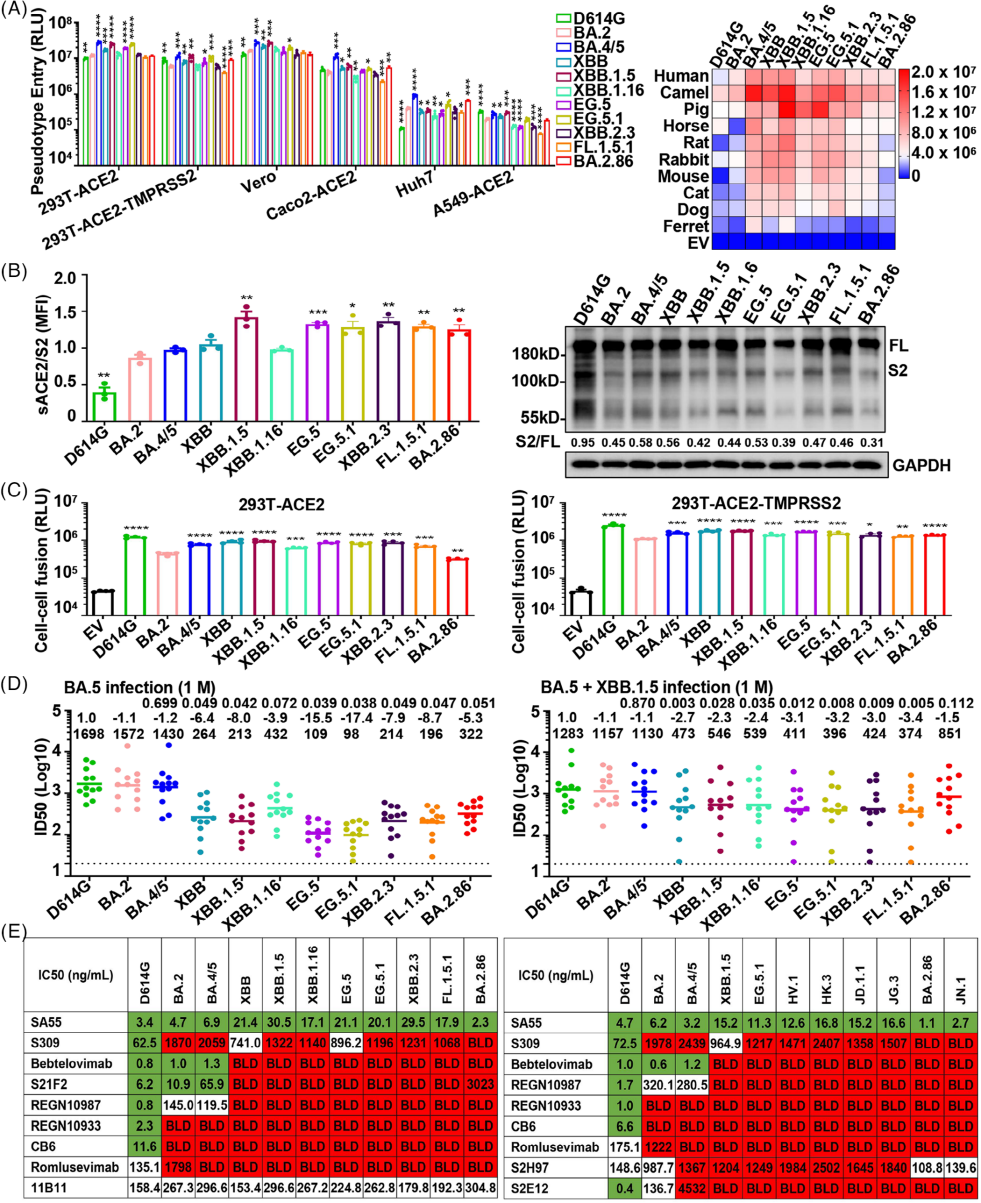
Antigenicity and infectivity of severe acute respiratory syndrome coronavirus 2 (SARS‐CoV‐2) Omicron subvariants EG.5.1, XBB.2.3, FL.1.5.1, and BA.2.86. (A) Infectivity of pseudoviruses carrying Omicron variants spike proteins against various susceptible cells (left panel) and 293T cells expressing 10 angiotensin‐converting enzyme 2 (ACE2) homologs (right panel). RLU, relative luminescence units. (B) Binding affinities (left panel) and proteolytic cleavage capacity (right panel) of newly emerging Omicron variants. MFI, mean fluorescence intensity; FL, full‐length; S2, S2 subunit. (C) Fusion activities mediated by variants spike proteins on 293T‐ACE2 or 293T‐ACE2‐TMPRSS2 cells after coculture for 12 h. (D) Half‐maximal inhibitory dilution (ID50) of serum samples obtained from participants with BA.5 breakthrough infection or BA.5 + XBB.1.5 breakthrough infection against the indicated SARS‐CoV‐2 variants. The fold changes of ID50 titers between variants and D614G pseudoviruses were presented above symbols. The dashed line indicates the initial dilution of serum samples. M, month. (E) Neutralizing activity of neutralizing mAbs against SARS‐CoV‐2 variants pseudovirus. Green, half‐maximal inhibitory concentration (IC50) < 100 ng/mL; Red, IC50 > 1000 ng/mL; BDL, below the detection limit. Experiments were performed in 3–4 replicates and repeated at least twice. One representative was shown with error bars indicating the standard error of the mean (SEM). Stars represent statistical differences of all SARS‐CoV‐2 subvariants compared to the BA.2 strain. P values are displayed as *p < 0.05, **p < 0.01, ***p < 0.001, and ****p < 0.0001. EV stands for empty vector.

To elucidate the impact of spike protein mutations on receptor engagement, we assessed their affinity for soluble recombinant ACE2 via flow cytometry. Analysis showed that Omicron subvariants' spike proteins have a higher affinity for human ACE2 than the D614G variant (Figure 1B, left panel). The affinity of BA.4/5, XBB, and XBB.1.16 spike proteins for human ACE2 was similar to that of BA.2, but XBB.1.5, EG.5, EG.5.1, and BA.2.86 were significantly higher, which may be a key factor in the increased transmissibility of these variants within human populations. Next, we analyzed the proteolytic process of the variant spike protein by western blot. The cleavage of spike protein was significantly reduced in all Omicron subvariants compared to D614G (Figure 1B, right panel). For BA.2 and related subvariants, including BA.4/5, XBB, XBB.1.5, XBB.1.16, EG.5, EG.5.1, XBB.2.3, FL.1.5.1, and BA.2.86, their protein cleavage capacity remained at low levels. This is important for understanding the pathogenicity and transmissibility of emerging Omicron variants.

Spike protein‐mediated fusogenicity is crucial for SARS‐CoV‐2 infectivity. Consistent with previous reports,^2^ all examined Omicron variants demonstrated diminished syncytium formation relative to the ancestral D614G (Figure 1C). Compared with BA.2, BA.2.86 showed lower fusion activity, whereas BA.4/5, XBB, XBB.1.5, XBB.1.16, EG.5, EG.5.1, XBB.2.3, and FL.1.5.1 manifested significantly higher fusogenicity. When 293T‐ACE2 overexpressing transmembrane protease serine 2 (TMPRSS2) was used as target cells, the fusion activity of these emerging Omicron subvariants showed a similar trend, except for BA.2.86, which showed significantly enhanced fusion activity. The enhanced fusion activity of BA.2.86 strain in TMPRSS2 overexpressed cells may be an important factor in the enhancement of viral infection.

We also investigated the selection of Omicron variant pseudoviruses entry pathways into host cells. We infected 293T‐ACE2‐TMPRSS2 cells with Omicron variant pseudovirus in the presence of Camostat (an inhibitor of serine proteases) and/or E64d (an inhibitor of cathepsins). SARS‐CoV‐2 D614G entered cells mainly through the TMPRSS‐dependent pathway, while Omicron was more dependent on the endocytic pathway (Figure S1A), which was consistent with previous studies.^4^ It has been reported that Omicron entry in human airway and intestinal organoid models relied on the expression of TMPRSS2 but not cathepsins.^5^ However, our experiments with Chloroquine (alkalization of phagolysosomes), Apilimod (a potent PIKfyve inhibitor), and E64d showed that all inhibitors reduced Omicron entry in a dose‐dependent manner, with consistent effects across variants (Figure S1B).

Mutations in SARS‐CoV‐2 spike proteins may modify viral antigenicity, potentially facilitating evasion from antibody‐mediated neutralization.^1^ Serum neutralizing activity was assessed in individuals with BA.5 or BA.5 + XBB.1.5 breakthrough infection after receipt of three doses of inactivated SARS‐CoV‐2 vaccine (Figure 1D). Approximately one month post‐BA.5 infection, the geometric mean titers for BA.2, BA.4/5, XBB, XBB.1.5, XBB.1.16, EG.5, EG.5.1, XBB.2.3, FL.1.5.1, and BA.2.86 were 1572, 1430, 264, 213, 432, 109, 98, 214, 196, and 322, respectively. Reassuringly, BA.2.86 was more potent than EG.5.1, XBB.2.3, and FL.1.5.1 in neutralizing human serum after BA.5 infection. Intriguingly, we observed enhanced serum‐neutralizing activity against the Omicron subvariants in participants reinfected with the XBB.1.5 strain following a BA.5 infection.

We also analyzed the neutralization susceptibility of Omicron subvariants to various therapeutic monoclonal antibodies (mAbs). Our findings indicate a significant reduction or complete loss of neutralization efficacy of most mAbs against Omicron subvariants (Figure 1E). S309 and S2H97 maintained partial neutralization activity against certain Omicron subvariants, but S309 was ineffective against BA.2.86 and JN.1. Remarkably, SA55 demonstrated effective neutralization across all SARS‐CoV‐2 variants. Additionally, mAb 11B11, targeting ACE2, exhibited robust neutralization capabilities against all Omicron strains, implying that such mAbs could possess broad‐spectrum antiviral properties.

Viral infectivity and immune resistance are pivotal risk attributes of newly emerging SARS‐CoV‐2 variants. Mutations in the Omicron spike protein significantly increase viral infectivity in cells expressing ACE2 homologs from non‐human animals, suggesting potential new animal reservoirs for Omicron and possibly accelerating its spread among humans. The spike protein's receptor‐binding domain (RBD) is instrumental in determining the host range for viral infection. Notably, XBB.1.5, EG.5, EG.5.1, XBB2.3, FL.1.5.1, and BA.2.86 exhibited stronger binding to ACE2 compared to the BA.2 strain, suggesting a link between their increased cellular infectivity and enhanced receptor binding affinity. It has been reported that mutations near the Furin cleavage site in Omicron variants may impact spike protein cleavage and membrane fusion.^2^ Our in vitro studies indicate that the fusogenicity mediated by Omicron subvariant spike proteins is generally weaker than the D614G strain. However, BA.4/5, XBB, XBB.1.5, XBB.1.6, EG.5, EG.5.1, XBB.2.3, and FL.1.5.1 exhibit stronger fusion than the original BA.2 strain, highlighting the need for further research on their infectivity and pathogenic potential.

Omicron variants are continually evolving under immune pressure, exhibiting enhanced immune evasion capabilities. Despite the robust immune escape potential of Omicron subvariants, our findings indicate that SA55, an antibody targeting the SARS‐CoV‐2 RBD, and 11B11, an antibody targeting the receptor ACE2, maintain effective neutralization against multiple Omicron subvariants. The substantial mutations in the SARS‐CoV‐2 BA.2.86 variant spike protein raised concerns regarding its ability to deeply evade antibody protection. Contrary to expectations, our study reveals that BA.2.86 does not demonstrate increased resistance to serum neutralization from individuals infected with BA.5 or XBB.1.5. Due to the dynamic nature of Omicron variant spike proteins, there is a key demand to intensify surveillance of Omicron variants and evaluate the effectiveness of therapeutic mAbs and vaccines against emerging SARS‐CoV‐2 variants.

## AUTHOR CONTRIBUTIONS

Haijun Tang and Yanhang Zhuo performed the experiments and Haijun Tang, Xiaohong Du wrote the manuscript; Haijun Tang and Xiaohong Du analyzed data; Xiaohong Du, Frank Xiao‐Feng Qin, and Yi Huang were responsible for the research design, strategy, and supervision. All authors have read and approved the final manuscript.

## CONFLICT OF INTEREST STATEMENT

The authors declare no conflict of interest.

## FUNDING INFORMATION

This work was supported by: The Chinese Academy of Medical Sciences Initiative for Innovative Medicine (2022‐I2M‐2‐004 and 2016‐I2M‐1‐005); CAMS Innovation Fund for Medical Sciences (CIFMS, 2023‐I2M‐2‐010); Joint Fund of Science and Technology Innovation of Fujian Province (2023Y9324); Medical Vertical Project of Fujian Province (2020CXB001); Joint Fund of Science and Technology Innovation of Fujian Province (2021Y9024); Key Project of Natural Science Foundation of Fujian Province (2022J02048); The Suzhou Municipal Key Laboratory (SZS2023005).

## ETHICS STATEMENT

This study was performed in strict accordance with human subject protection guidance proved by the Research Ethics Committee of Fujian Provincial Hospital (number: K2023‐03‐011).

## Supporting information

Supporting Information

## Data Availability

The data that support the findings of this study are available from the corresponding author upon reasonable request.
