# Supplementary material for: Antigenicity and infectivity of severe acute respiratory syndrome coronavirus 2 Omicron subvariants EG.5.1, XBB.2.3, FL.1.5.1, and BA.2.86
Source: MedComm (2020). 2024 Jun 8;5(6):e589. doi: 10.1002/mco2.589 (PMC11161722; doi:10.1002/mco2.589)
Supplement: Supplementary file 1 — Supporting Information [file MCO2-5-e589-s001.doc]

Supplementary Information

**Antigenicity and infectivity of SARS-CoV-2 Omicron subvariants EG.5.1, XBB.2.3, FL.1.5.1, and BA.2.86**

Haijun Tang^1,2,3^**^#^**, Yanhang Zhuo^1,2^**^#^**, Xiaohong Du^3^*, F. Xiao-Feng Qin^3^*, Yi Huang^1,2^*

^1^Shengli Clinical Medical College of Fujian Medical University, Fuzhou 350001, China.

^2^Center for Experimental Research in Clinical Medicine, Central Laboratory, Fujian Provincial Hospital, Fuzhou 350001, China.

^3^National Key Laboratory of Immunity and Inflammation, Suzhou Institute of Systems Medicine, Chinese Academy of Medical Sciences & Peking Union Medical College, Suzhou, 215123, China.

**Materials and Methods**

**Serum samples**

Blood samples were collected from participants with Omicron BA.5 breakthrough infection (1 month) or Omicron BA.5 + XBB.1.5 breakthrough infection (1 month) after receipt of three doses of inactivated SARS-CoV-2 vaccine.

**Monoclonal antibodies and inhibitors**

Monoclonal antibodies (mAbs) were synthesized by AtaGenix based on the published sequences in the Protein Data Bank. Among them, neutralizing mAbs SA55, S309, Bebtelovimab, S21F2, REGN10987, REGN10933, CB6, Romlusevimab, S2H97, and S2E12 target the RBD domain of SARS-CoV-2 spike protein ^1-3^. In contrast, mAb 11B11 targets ACE2, the receptor for SARS-CoV-2 ^4^. Small molecule inhibitors including Camostat (an inhibitor of serine proteases) ^5^, E64d (an inhibitor of cathepsins) ^5^, Chloroquine (alkalization of phagolysosomes) ^6^, and Apilimod (a potent PIKfyve inhibitor) ^7^ were purchased from MedChemExpress.

**Cell lines**

Caco-2, 293T, Huh7, A549 and Vero cells were obtained from the American Type Culture Collection. 293T-ACE2, 293T-ACE2-TMPRSS2, Caco2-ACE2 and A549-ACE2 cells were constructed by lentivirus-mediated gene transduction.

**Production of SARS-CoV-2 variants pseudoviruses**

The pcDNA3.1 plasmid encoding SARS-CoV-2 variants spike protein was synthesized by Genscript Biotechnology. Pseudoviruses harboring spike proteins of SARS-CoV-2 variants were generated in the background of vesicular stomatitis virus (VSV) as described previously ^8^.

**Flow cytometric analysis of soluble ACE2 binding to variants spike proteins.**

The binding activity of SARS-CoV-2 variants spike proteins to soluble ACE2 by flow cytometry according to previously reported ^9^. Binding activity was determined by mean fluorescence intensity (MFI) and normalized by cell surface expression of spike proteins (sACE2/S2). All MFI values were weighted by multiplying by the number of positive cells.

**Western blot analysis of Omicron variants spike proteins cleavage**

We transfected the expression plasmids encoding the variants spike proteins in 293T cells. At 36 h after transfection, we lysed cells with RIPA buffer. Lysates were diluted in 5-fold sample buffer, boiled for 10 min, and then analyzed using western blot. Protein detection was performed using the following antibodies: mouse anti-SARS-CoV-2 spike (Genetex), GAPDH monoclonal antibody (Proteintech), horseradish peroxidase-linked anti-mouse IgG antibody (Cell Signaling Technology).

**Cell-cell fusion assay**

Prior to cell-cell fusion assay, we detected the expression of variants spike proteins in 293T cells by Western Blot to ensure their expression levels were similar. Cell-cell fusion mediated by ACE2 and variant spike proteins was detected using the T7 polymerase reporter system as previously reported ^8^.

**Pseudovirus neutralization assay**

Neutralizing mAbs were serially diluted at a starting concentration of 10 μg/mL (Bebtelovimab, 1 μg/mL) and then incubated with 1000 TCID50 pseudoviruses for 1 h at 37°C. To test the neutralizing activity of vaccine sera, serial 5-fold dilution of samples were prepared with the initial dilution of 1:20. The mixture was inoculated into 293T-ACE2 cells and incubated at 37°C for 24 h. Neutralization activity was determined by measuring luciferase activity in cell lysates ^8^.

**Statistical Analysis**

GraphPad Prism 8 was used for statistical analysis and plotting of the data. Data were expressed as mean ± SEM. The Wilcoxon paired signed rank test was used to evaluate the neutralizing activity of sera against variant pseudoviruses. A p-value of less than 0.05 was considered statistically significant (p < 0.05 [*], p < 0.01 [**], p < 0.001 [***], p < 0.0001 [****]).

**References**

1. Cao Y, Yisimayi A, Jian F, et al. BA.2.12.1, BA.4 and BA.5 escape antibodies elicited by Omicron infection. *Nature*. 2022;

2. Shi R, Shan C, Duan X, et al. A human neutralizing antibody targets the receptor-binding site of SARS-CoV-2. *Nature*. 2020;584(7819):120-124.

3. de Campos-Mata L, Trinité B, Modrego A, et al. A monoclonal antibody targeting a large surface of the receptor binding motif shows pan-neutralizing SARS-CoV-2 activity. *Nature communications*. 2024;15(1):1051.

4. Du Y, Shi R, Zhang Y, et al. A broadly neutralizing humanized ACE2-targeting antibody against SARS-CoV-2 variants. *Nature communications*. 2021;12(1):5000.

5. Hoffmann M, Kleine-Weber H, Schroeder S, et al. SARS-CoV-2 Cell Entry Depends on ACE2 and TMPRSS2 and Is Blocked by a Clinically Proven Protease Inhibitor. *Cell*. 2020;181(2):271-280.e8.

6. Wang M, Cao R, Zhang L, et al. Remdesivir and chloroquine effectively inhibit the recently emerged novel coronavirus (2019-nCoV) in vitro. *Cell research*. 2020;30(3):269-271.

7. Su J, Zheng J, Huang W, et al. PIKfyve inhibitors against SARS-CoV-2 and its variants including Omicron. *Signal transduction and targeted therapy*. 2022;7(1):167.

8. Du X, Tang H, Gao L, et al. Omicron adopts a different strategy from Delta and other variants to adapt to host. *Signal transduction and targeted therapy*. 2022;7(1):45.

9. Wang R, Zhang Q, Ge J, et al. Analysis of SARS-CoV-2 variant mutations reveals neutralization escape mechanisms and the ability to use ACE2 receptors from additional species. *Immunity*. 2021;54(7):1611-1621.e5.

**Supplementary Figure and Figure Legend**

**
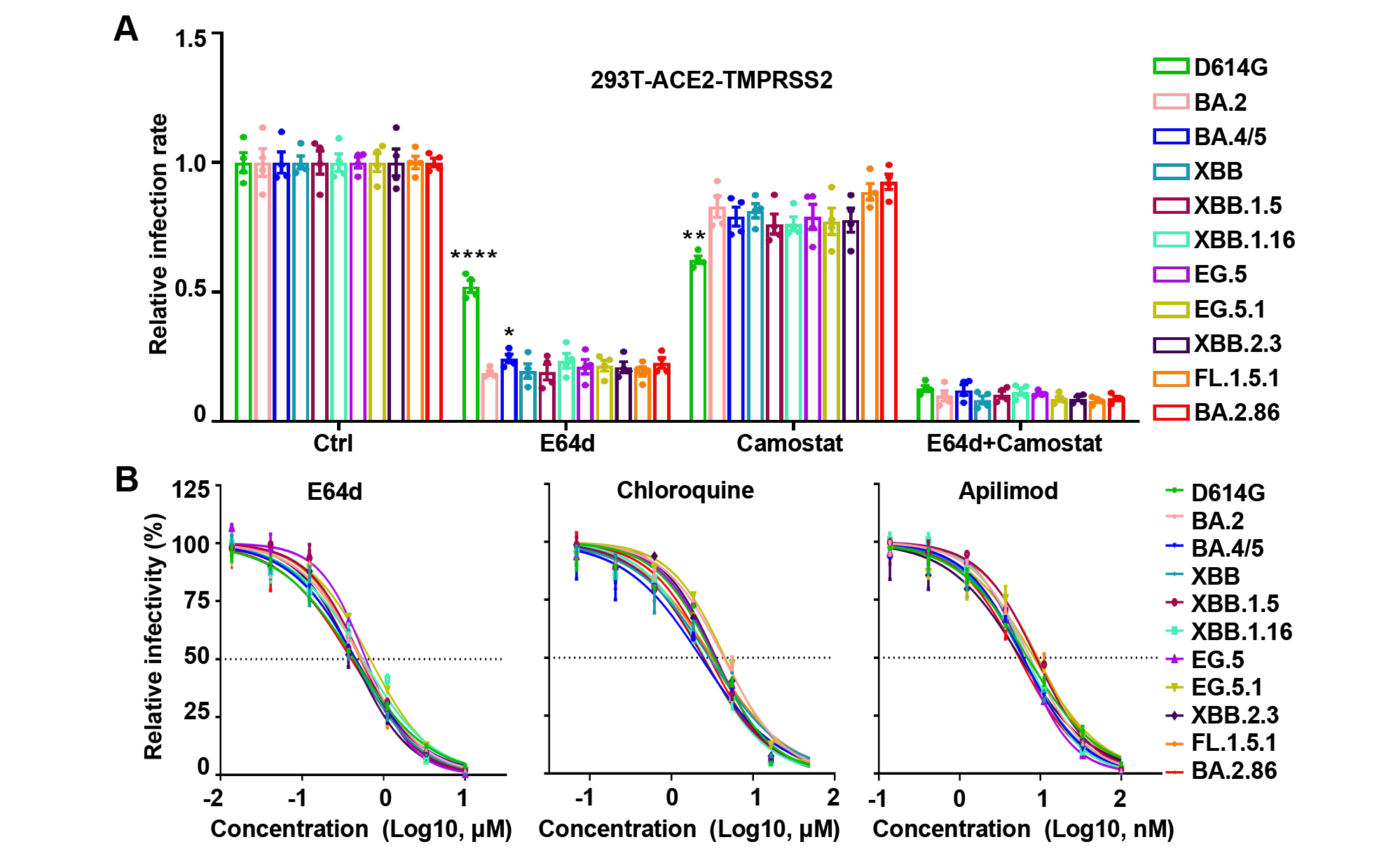
**

**Figure S1. Effect of endocytosis or protease inhibitors on SARS-CoV-2 variants pseudovirus entry. (A)** The effect of E64d and Camostat treatment on variant pseudovirus infection. 293T-ACE2-TMPRSS2 cells were pretreated with E64d, Camostat, or both for 2 h, and then the invasion pathway of the variant pseudovirus was examined. **(B)** Effect of cathepsin inhibitor or endocytosis pathway inhibitor on the entry of SARS-CoV-2 variants pseudovirus into 293T-ACE2 cells.
